# Development of a Conversational Agent for Individuals Ambivalent About Quitting Smoking: Protocol for a Proof-of-Concept Study

**DOI:** 10.2196/44041

**Published:** 2023-03-31

**Authors:** Uma S Nair, Karah Greene, Stephanie Marhefka, Kristin Kosyluk, Jerome T Galea

**Affiliations:** 1 College of Nursing University of South Florida Tampa, FL United States; 2 School of Social Work, College of Behavioral and Community Sciences University of South Florida Tampa, FL United States; 3 Mental Health Law and Policy, College of Behavioral and Community Sciences University of South Florida Tampa, FL United States; 4 Department of Global Health and Social Medicine Harvard School of Medicine Boston, MA United States

**Keywords:** cigarettes, conversational agent, mhealth, smoking cessation

## Abstract

**Background:**

Cigarette smoking is the leading preventable cause of disease and death in the United States. Despite the availability of a plethora of evidence-based smoking cessation resources, less than one-third of individuals who smoke seek cessation services, and individuals using these services are often those who are actively contemplating quitting smoking. There is a distinct dearth of low-cost, scalable interventions to support smokers not ready to quit (ambivalent smokers). Such interventions can assist in gradually promoting smoking behavior changes in this target population until motivation to quit arises, at which time they can be navigated to existing evidence-based smoking cessation interventions. Conversational agents or chatbots could provide cessation education and support to ambivalent smokers to build motivation and navigate them to evidence-based resources when ready to quit.

**Objective:**

The goal of our study is to test the proof-of-concept of the development and preliminary feasibility and acceptability of a smoking cessation support chatbot.

**Methods:**

We will accomplish our study aims in 2 phases. In phase 1, we will survey 300 ambivalent smokers to determine their preferences and priorities for a smoking cessation support chatbot. A “forced-choice experiment” will be administered to understand participants’ preferred characteristics (attributes) of the proposed chatbot prototype. The data gathered will be used to program the prototype. In phase 2, we will invite 25 individuals who smoke to use the developed prototype. For this phase, participants will receive an overview of the chatbot and be encouraged to use the chatbot and engage and interact with the programmed attributes and components for a 2-week period.

**Results:**

At the end of phase 1, we anticipate identifying key attributes that ambivalent smokers prefer in a smoking cessation support chatbot. At the end of phase 2, chatbot acceptability and feasibility will be assessed. The study was funded in June 2022, and data collection for both phases of the study is currently ongoing. We expect study results to be published by December 2023.

**Conclusions:**

Study results will yield a smoking behavior change chatbot prototype developed for ambivalent smokers that will be ready for efficacy testing in a larger study.

**International Registered Report Identifier (IRRID):**

DERR1-10.2196/44041

## Introduction

Cigarette smoking continues to be the leading preventable cause of disease and death globally and in the United States [1]. Cigarette smoking kills more than 480,000 Americans each year and results in more than US $240 billion in health care spending and nearly US $372 billion in lost productivity [2]. Thus, promoting smoking behavior change with the long-term goal of achieving successful cessation is critical to reducing the public health impact caused by tobacco use. Within the United States, the past few decades have seen a groundswell of evidence-based interventions to quit smoking. These have included a wide range of strategies such as mobile health (mHealth) apps [3,4], availability and increased access to effective pharmacotherapy for cessation [5], SMS text messaging support [6,7], evidence-based behavioral counseling [8], and comprehensive state-based programs, such as tobacco quitlines [9,10]. However, despite their widespread availability, less than one-third of people who smoke cigarettes actively avail themselves to cessation strategies [11]. For example, tobacco quitlines—free evidence-based smoking cessation programs providing telephone-based behavioral counseling combined with cessation pharmacotherapy available in all 50 US states—are used by less than 3% of people who smoke [12]. One potential reason for the low uptake of existing services for cessation may be that strategies to promote cessation differ according to an individual’s readiness and motivation to quit [13]. Although smokers actively committed to quitting respond positively and engage with cessation services, ambivalent smokers (ie, those not actively seeking treatment) may benefit from approaches that promote modest changes or “behavioral *nudges*” toward smoking behavior change (but not cessation) [14,15]. Most cessation research is focused on those expressing an intention to quit, and research on promoting behavior change in ambivalent smokers (not actively thinking about quitting) is scant.

A conversational agent or “chatbot” is an inexpensive and scalable approach to deliver personalized health information in a human-like manner. Common in the consumer-service sector, chatbots are increasingly being deployed in the health sector to provide education, support, service navigation, and interventions for a range of health issues, from COVID-19 to asthma [16,17]. Human-delivered interventions for smoking behavior change are effective but are resource-intensive, lack scalability, and can be expensive to implement. Chatbots have the intuitive appeal of being low-cost automated interventions that can widely reach ambivalent smokers. Moreover, chatbots can provide “virtual accompaniment” at the frequency and duration preferred by the smoker until the motivation to quit arises, at which time they can be swiftly connected to an existing evidence-based cessation intervention. A few studies on chatbots for smoking behavior change target participants actively intending to quit smoking [18-20] and, while important, only address the tip of the iceberg in terms of reaching the smoking population. Since motivation to quit smoking is dynamic and occurs along a continuum [21], strategies to engage ambivalent smokers may be most helpful if they are tailored to accommodate smokers’ changing preferences and are responsive to rapid changes in motivation (eg, connecting smokers to quitlines when ready to quit), while focusing on evidence-based strategies that have been effective for smoking reduction leading to cessation (eg, quit tips, self-monitoring, and tracking savings). Since interventions to assist smokers ambivalent about quitting are in their infancy, little is known about key elements of a chatbot-based smoking behavior change intervention tailored for this target population.

To overcome these gaps in the field, the goal of our study is to test a proof-of-concept of a novel chatbot to support and engage smokers who are not actively engaged in a quit attempt (ambivalent smokers). We propose accomplishing our aims in 2 phases. In phase 1, we will identify the key attributes that will guide the development of the chatbot prototype. We will survey ambivalent smokers to determine their preferences and priorities for a smoking cessation support chatbot. Using conjoint analysis, we will systematically elicit participant preferences for the proposed chatbot prototype (see details below). Grounded in macroeconomic principles [22], conjoint analysis is a quasi-experimental approach that decomposes a product or service into its key attributes, then poses the attributes to patients to understand patient-determined values for each attribute [23,24]. These preferences will be programmed into the chatbot prototype. In phase 2, we will invite participants to use the prototype over a 2-week period to assess the feasibility and acceptability of the developed chatbot prototype.

## Methods

### Study Overview

To develop the chatbot prototype (phase 1), we will recruit 300 participants who identify as current cigarette smokers to complete a web-based survey. The survey will collect demographics, smoking behavior history, and e-cigarette use, and solicit preferences for the most important features of the proposed chatbot. The top 5 preferred attributes will be used to design and develop the chatbot prototype. In phase 2, 25 participants will be recruited to engage with the developed prototype for a 2-week period. Preliminary acceptability and feasibility data will be collected via validated questionnaires at the end of 2 weeks. For both study phases, participants will be recruited using an established survey panel (Prolific). Study eligibility criteria are as follows: older than 18 years, self-report any smoking in the last 7 days, and no current intention to quit smoking in the next 30 days (score of ≥5 on a 10-point Likert scale assessing likelihood to quit smoking).

### Ethics Approval

All study procedures have been approved by the University of South Florida’s Institutional Review Board (IRB # 004183). For phase 1, the data are deidentified with participants only providing a prolific ID. For phase 2, participant names and phone numbers will be stored on a password-protected computer only accessible to the study staff. All participants will be identified by a study ID and any identifying information will be removed from interview transcripts prior to beginning qualitative data analysis. Participants will be compensated for both phases of the study. Participants will receive US $6 for completing phase 1 and US $70 for completing phase 2.

### Phase 1: Development of Chatbot Prototype

#### Overview

This phase is designed as a cross-sectional survey to develop a prototype of the proposed chatbot by assessing preferred attributes in a sample of individuals ambivalent about quitting smoking. To do so, 300 participants have been identified who will be invited to complete a web-based survey. Participants will be recruited using the internet and an established study panel (Prolific). After completing a web-based informed consent, participants will provide information on demographics and smoking history, after which they will be provided with a brief explanation of chatbots (what they are and how they work). They will also be asked about their previous and current experience with chatbots and interest in a chatbot designed to support people who may decide to quit smoking in the future.

**Figure 1:**
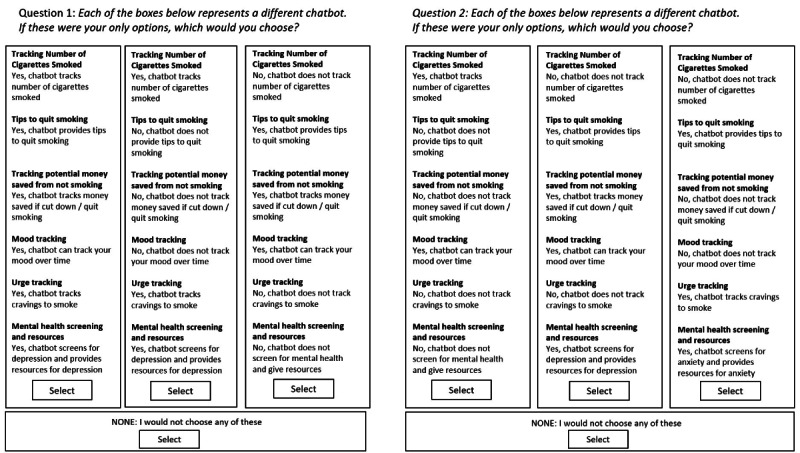
Examples of a conjoint analysis task.

Next, we will administer a choice-based conjoint (CBC) analysis experiment to understand participants’ preferences for a smoking cessation chatbot. Using Sawtooth Software’s web-based choice analytics survey platform, we will construct hypothetical smoking cessation chatbot “scenarios” comprising the following six attributes (ie, potential chatbot features) with varying levels: (1) number of cigarettes smoked per day tracked (yes or no), (2) smoking quit-tips provided (yes or no), (3) money saved by reducing smoking tracked (yes or no), (4) mood tracking (yes or no), (5) smoking cravings tracked (yes or no), and (6) mental health screening and resource provision (only depression screening, only anxiety screening, or no mental health screening). These attributes were chosen a priori based on published smoking cessation literature identifying evidence-based strategies to promote and support smoking behavior change. The sixth attribute, mental health screening, was added given the comorbidity between smoking and mental health [25,26]. In the CBC experiment, participants will be presented with a series of tasks; each task will display 3 different chatbot configurations composed of identical attributes but differing combinations of attribute levels. A total of 10 tasks will be presented to participants. Each question will contain 3 panels that present a combination of the attributes and associated scenarios, and participants will be asked to choose the chatbot configuration they would most prefer (ie, the chatbot that contains the combination of chatbot attribute levels most desired). Participants may also choose “none” if no chatbot configuration presented is desirable. Figure 1 displays examples of the CBC tasks. Finally, the desired frequency of chatbot messages will be assessed, and at the end of the survey, interest in participating in phase 2 will be elicited.

#### Measures

For phase 1, assessments will include demographics (eg, age, sex, gender identity, and education) and smoking history (age of smoking initiation and number of previous quit attempts in the past year) [27]. Nicotine dependence will be measured by the Fagerstrom Test of Nicotine Dependence [28], which is a validated and ubiquitous instrument assessing dependence on nicotine. Smoking cessation self-efficacy will be measured using the validated Smoking Abstinence Self-Efficacy Questionnaire [29]. This 12-item questionnaire measures an individual’s self-reported confidence in resisting the urge to smoke in various settings and conditions. Given the high prevalence of e-cigarette use among individuals who smoke [30], current e-cigarette use will also be assessed [31]. In addition to these measures, we will also collect information on current use and frequency of marijuana or cannabis [32] and alcohol use [33].

#### Data Analysis

Summary statistics (mean [SD] or frequency [%]) will be calculated for demographic and smoking characteristics. For the CBC data analysis, also completed using Sawtooth Software [34], utility scores will first be computed to determine the relative desirability of each level within each attribute. These data will inform which of the chatbot feature levels were most desired (eg, whether a cigarette tracking feature is desired). Next, the relative attribute importance scores (ie, the relative impact of each attribute in respondent choices) will be computed; these data will indicate the order of importance of each of the 6 chatbot features (most important to least important). We will also compute the 95% CIs for each of the attribute scores. Finally, CBC data will be examined for variance by select sociodemographic variables (eg, sex, gender, education, and number of quit attempts) to determine possible chatbot preference differences by population segments. The combined data will inform the prioritization of chatbot features programmed into the prototype chatbot tested in phase 2.

### Phase 2: Acceptability and Feasibility Testing of the Chatbot Prototype

#### Overview

For phase 2, 25 eligible individuals expressing interest to participate at the end of phase 1 will be recruited to engage with the developed chatbot prototype. Participants will be contacted by the study staff to explain study procedures and complete informed consent, following which the participant will receive a link to complete a baseline survey. Following completion of the survey, they will be scheduled for an appointment to participate in a 45- to 60-minute interview conducted via secure videoconferencing. During this time, study staff will share a link with the participants to activate the chatbot prototype. An overview of the chatbot will be provided, following which participants will verbalize their thoughts and reactions while interacting with the chatbot, based on the think-aloud usability testing technique [32], and provide initial feedback while interacting with the chatbot. The interview will be audio and video recorded and participant interactions with the chatbot will be observed. At the end of this session, participants will be encouraged to use the chatbot over a 2-week period, engaging and interacting with the programmed attributes and components. To increase retention during this period, data entry and chatbot engagement will be monitored on a frequent basis, and all participants will receive daily reminders to engage with the chatbot. At the end of the 2-week period, participants will complete acceptability and feasibility assessments, provide feedback to open-ended questions to identify any aspect of the chatbot that was confusing, what they liked or disliked, perceived helpfulness of the chatbot, feedback on features they would like included, and aspects that would help keep them engaged with the chatbot. Participants will receive a total of US $70 for phase 2 (US $20 for the interview and US $50 after completion of the follow-up surveys).

#### Measures

In addition to the measures used in phase 1, phase 2 measures will include intervention acceptability using the Acceptability of Intervention Measure, Intervention Appropriateness Measure, and Feasibility of Intervention Measure [33], as well as intervention usability using Health Information Technology Usability Evaluation Scale [35].

#### Data Analysis

Quantitative data will be cleaned and analyzed to generate summary tables. We will limit analysis to only include descriptives due to the small sample size, which prevents tests of association. Qualitative data (ie, recorded transcripts from the videos during which participants interact with the chatbot and the qualitative interviews after interacting with the chatbot) will first be transcribed verbatim, resulting in text transcripts. Next, the transcripts will be loaded into the qualitative software, Dedoose [36], and analyzed using a framework analysis approach [37]. Transcripts will be read in their entirety and coded using a preliminary codebook derived from the interview guide, adding de novo codes as new themes are detected. Reports will be generated for all codes and related text extracted into tables for deeper analysis, during which cross-cutting themes will be identified and reported. The COREQ (Consolidated Criteria for Reporting Qualitative Data) checklist [38] will be completed to enhance data rigor and methodological transparency [28-31].

## Results

The study was funded in June 2022, and regulatory processes and approval were completed by August 2022. Data collection for the study is currently ongoing, and we anticipate publication of expected outcomes by December of 2023. In phase 1, the methodology described above will result in the identification of key attributes that participants report a preference for in a smoking cessation support chatbot. These data will be used to program a chatbot prototype. Using SmartBot360 [39], a “point-and-click” chatbot development software, a prototype smoking cessation support chatbot will be programmed that contains the top preferences and features as expressed by the participants in phase 1. In addition to the attributes, information on existing evidence-based smoking cessation resources will also be programed to gauge participant preferences for existing evidence-based cessation resources. These will include, but not be limited to, links to sign up for free SMS text messaging services (eg, smokefree TXT) or mHealth quit apps (eg, QuitGuide), connecting to smoking cessation experts (eg, National Cancer Institute’s LiveHelp or a state quitline), and information on pharmacotherapy.

At the end of phase 2, we will obtain participant data on chatbot acceptability and feasibility.

## Discussion

The goal of our study is to develop and pilot-test for feasibility and acceptability of a novel chatbot prototype to support and engage smokers who are not actively engaged in a quit attempt (ambivalent smokers). The study will test a proof-of-concept of the chatbot, and the anticipated main result of our project is a pilot-tested conversational agent ready to be tested for efficacy in a larger study. To our knowledge, this is the first study to develop a conversational agent (chatbot) for ambivalent smokers using a novel methodology.

Interventions to engage individuals ambivalent about quitting smoking are an unexplored area in smoking cessation research [11]. This study will begin an innovative line of research on strategies to optimize and personalize smoking behavior change services for individuals ambivalent about quitting smoking. Of specific mention is the use of conjoint analysis as an innovative strategy to assess and incorporate user preferences in the development of an mHealth intervention. Methodologies to systematically assess and incorporate individual preferences in the delivery of health care services are in their infancy [40], and little is known about the preferences of ambivalent smokers for chatbots as they navigate the motivation to quit continuum. While conjoint analysis has been increasingly used in the health sector, including in the smoking cessation field [41,42], to our knowledge, no mHealth interventions for smoking behavior change have used this methodology for design and implementation. Our investigative team has successfully used conjoint analysis for other health issues, including determining consumer preferences for oral and topical HIV chemoprophylaxis [43,44]. If acceptable in this pilot study, our chatbot could be a low-cost and low-resource intervention that fills a much-needed gap in the field.

The study has a few limitations. First, the study design does not lend itself to testing the effects of the chatbot on smoking behavior change outcomes. However, given the dearth of research in this area, the development of the chatbot and acceptability and feasibility testing are the first steps. At the end of the study, we will have a developed chatbot that will be ready for efficacy testing in a larger randomized controlled trial. Next, our participant recruitment methodology uses a web-based survey panel, which may reduce generalizability. While such survey panels use purposive sampling to gain a representative sample of participants [45], we anticipate using a combination of social media, community-based recruitment efforts, and web-based recruitment panels for larger efficacy trials, which will increase the generalizability of our results.

Our intervention that uses a chatbot to promote smoking behavior change in a sample of ambivalent smokers fills a distinct gap in the field. Our model is a scalable and pragmatic model that can be adopted by cessation services (quitlines) and is designed for easy accessibility to the broader population of individuals who are not actively contemplating quitting smoking. It could additionally serve as a tool for health care providers as they recommend quitting smoking resources to their patients. In the long run, our study design and methodology can be used to develop chatbots that can increase access to other evidence-based interventions within the context of substance use disorders (eg, cannabis and opioids) or compulsive behaviors (eg, eating disorders and gambling).

## Abbreviations

CBC: choice-based conjoint
COREQ: Consolidated Criteria for Reporting Qualitative Data
mHealth: mobile health

## Data Availability

The data sets generated and analyzed during this study will be available from the study investigators (USN and JTG) on reasonable request.
